# Public mental health services in Southern China and related health outcomes among individuals living with severe mental illness

**DOI:** 10.1186/s41256-024-00363-0

**Published:** 2024-08-29

**Authors:** Dan Qiu, Yilu Li, Shuiyuan Xiao, Liang Zhou, Lianzhong Liu, Huiming Liu, Feihong Gao, Qiuyan Wu, Yanni An, Zixuan Tang

**Affiliations:** 1 Department of Social Medicine and Health Management, Xiangya School of Public Health, Central South University, 110 Xiangya Road, Changsha, 410078 Hunan China; 2 Mental Health Institute, Second Xiangya Hospital, Central South University, Changsha, Hunan China; 3 The Affiliated Brain Hospital of Guangzhou Medical University, Guangzhou, Guangdong China; 4 Wuhan Mental Health Center, Wuhan, Hubei China; 5 Shenzhen Kangning Hospital, Shenzhen, Guangdong China; 6 Shenzhen Mental Health Center, Shenzhen, Guangdong China; 7 The Ninth Hospital of Changsha, Changsha, Hunan China

**Keywords:** Mental health service, Severe mental illness, Contact coverage, China

## Abstract

**Background:**

Although national policies in China are comprehensive and instructive, a wide disparity exists between different cities. The current status of public mental health services by region in China remains unclear. This study aimed to investigate policies related to public mental health services, the contact coverage of public mental health services and outcomes of service users.

**Methods:**

A cross-sectional study was carried out in Southern China, between April 2021 and March 2022.  Considering the geographical location, socioeconomic development levels, and prevalence of severe mental illness, four cities including Wuhan, Changsha, Guangzhou, and Shenzhen were selected. Relevant service providers were asked to report data on mental health policies and facility-related information, including mental health resources, registration rates of patients, management rates of patients, and medication rates of patients. Eligible patients were invited to report service user-related data, including contact coverage of public mental health services and their outcomes. SPSS 26.0 was used for data analysis.

**Results:**

The four cities in Southern China have made different efforts to develop public mental health services, primarily focusing on socio-economically disadvantaged individuals. Community health centers in Guangzhou and Shenzhen reported having more professional human resources on mental health and higher mental health budgets for patients. The contact coverage rates of most public mental services were higher than 80%. Patients in Changsha (B = 0.3; 95%*CI:* 0.1–0.5), Guangzhou (B = 0.2; 95%*CI:* 0.1–0.3), and Shenzhen (B = 0.3; 95%*CI:* 0.1–0.4) who received social medical assistance services reported higher levels of medication adherence. Patients in Wuhan (B = -6.5; 95%*CI:* -12.9--0.1), Guangzhou (B = -2.8; 95%*CI:* -5.5--0.1), and Shenzhen who received community-based rehabilitation services reported lower levels of disability (B = -2.6; 95%*CI:* -4.6--0.5).

**Conclusions:**

There have been advances in public mental health services in the four Southern cities. The contact coverage rates of most public mental health services were higher than 80%. Patients’ utilization of public mental services was associated with better health outcomes. To improve the quality of public mental health services, the government should try to engage service users, their family members, and supporters in the design, delivery, operationalization, and evaluation of these public mental health services in the future.

**Supplementary Information:**

The online version contains supplementary material available at 10.1186/s41256-024-00363-0.

## Introduction

The burden of mental disorders continues to increase, with significant impacts on health, social, and economic aspects worldwide [1]. From 2011 to 2030, the cumulative global impact of mental disorders in terms of lost economic output will amount to $16.3 trillion [2]. Although mental disorders are significant contributors to the global health burden, the global median of government health expenditure allocated to mental health is less than 2% [3]. More than two out of three people with severe mental illnesses (SMI) in the world do not receive specialist mental health care [4], and many individuals affected in low- and middle-income countries do not even have access to treatment [4].

To close the gap in access to mental health services, several global initiatives call on countries across the world to provide comprehensive, integrated and responsive mental health and social care services in community-based settings [2, 3]. As one of the countries with a high burden of mental disorders [5], the Chinese government was eager to establish a community-based comprehensive mental health system as early as 20 years ago [6, 7], emphasizing the urgent need to improve public mental health services. In 2004, the national project – ‘Central Government Support for the Local Management and Treatment of Severe Mental Illnesses Project’, was initiated to integrate mental health services in China. This program was developed as part of the National Basic Public Health Services Program in 2009, now known as ‘The National Management and Intervention Program for Severe Mental Illness (NMIPSMI)’ [8]. The NMIPSMI program has developed a service model that enables the transfer of mental health care from specialized psychiatric hospitals to community settings, linking provincial and district hospitals to township or neighborhood-level health institutions, which integrate public mental health services into the community [8].

Between 2011 and 2018, the Chinese central government published several comprehensive guidelines to implement public mental health services [9–11]. Local governments were required to provide follow-up services and physical examinations for registered individuals with SMI (schizophrenia, schizoaffective disorder, paranoid psychosis, bipolar disorder, psychotic disorder due to epilepsy, mental retardation with psychotic symptoms) [6]. For patients who were socio-economically disadvantaged, local governments were required to provide social medical assistance, such as medication and hospitalization services [7]. With the development of the integrated mental health system, local governments are now also required to provide outpatient and rehabilitation services. However, a wide difference exists between areas in terms of social and economic aspects, local policies between governments are often inconsistent, and the progress of policy implementation between cities is also different [7]. The current status of public mental health services by region remains unknown.

Another problem is that although the integrated mental health services model has been developed since 2004, the services provided have not yet been evaluated because of a complex set of barriers [7]. For example, outcome evaluation was not incorporated into the initial design of the programs, making it methodologically challenging to conduct evaluation research [12]. Besides, researchers have no access to administrative data. No effective quality-control measures used for data quality is also a concern [7, 12]. Currently, 6.5 million individuals living with SMI are involved in the integrated healthcare system, seeking public mental health services provided by primary health centers [13]. However, no study has ever reported service-related data from the perspective of service users [14]. This study aimed to describe (a) policies related to public mental health services released in different cities; (b) resources for public mental health services in different health centers; and (c) the contact coverage of public mental health services and outcomes of service users.

## Methods

### Ethics statement

This study was approved by the Human Research Ethics Committee (XYGW-2021–41). Written informed consent was obtained before interviews were conducted.

### Procedures

This was a cross-sectional study, which was carried out between April 2021 and March 2022. Considering the geographical location, socioeconomic development levels, and prevalence of SMI, four cities in Southern China (Wuhan, Changsha, Guangzhou, and Shenzhen) were selected. To collect policy-related data, all administrative districts of the four cities were included as our sampling frame, and two districts were randomly selected in each city (a total of 8 districts in the four cities were selected, see Table S1 for the details). One relevant member in each included city was asked to report city-related policies, and one relevant member in each included district was asked to report district-related policies. To collect institution-related data, 50% of the community health centers in each district (63/122) were randomly selected to participate in our program, with 45 centers (71.4%) finally involved. One relevant staff member in each included community health center was asked to report institution-related data. In total, 57 staff in different agencies were enrolled. The inclusion criteria of different staff were presented in Table S1 and S2.

To collect data related to service users, eligible patients and their caregivers in the 45 included community health centers (*n* = 1880) were invited to their affiliated community health centers, where trained researchers conducted face-to-face interviews. Finally, 972 patients were enrolled. Participants who completed the questionnaire were reimbursed with RMB 20 yuan in cash. See Figure S1 and Table S2 for the details.

### Collection on policy-related data and institution-related data

The included staff in different agencies were asked to report data related to public mental health services. This included information on mental health policy documents issued by the local governments, data on mental health resources, registration rates of patients, management rates of patients, medication rates of patients. See Table S2 for the details.

### Measures for patient-related data

#### Contact coverage of public mental health services

Contact coverage of public mental health service was defined as a patient’s use of at least one public mental health service provided by the community health center or their cooperative hospital. This study had seven outcome variables specific to the contact coverage of public mental health services over the past year.

Patients were asked six questions: over the past year, a) “have you utilized the follow-up service offered by the community health center? if so, what is the frequency?”; b); “have you utilized the physical examination services offered by the community health center?”; c) “have you utilized the medicine distribution service offered by the community health center or their cooperative hospital?”; d) “have you utilized the outpatient service offered by the community health center or their cooperative hospital?”; e) “have you utilized the inpatient service offered by the community health center or their cooperative hospital?”; f) “have you utilized the rehabilitation services offered by the community health center or their cooperative hospital?” The response options for these questions were “Yes”, “No” and “Not clear”.

In addition, guardians or family caregivers of the included patients were asked for one question: g) Over the past year, “have you or your family ever received a guardian allowance offered by the community health center or their cooperative hospital?”.

### Outcomes of patients

Disability was self-assessed by the 12-item World Health Organization Disability Assessment Schedule 2.0 (WHODAS 2.0) [15] to measure disability and functional impairment. Functioning was clinician-assessed by the Global Assessment of Functioning (GAF) to measure a person’s psychological, social, and occupational functioning ranging from 1 to 100 [16]. Quality of life was measured using the first two general questions from the 14-item World Health Organization Quality of Life Brief Scale (WHOQOL-BREF) [17]. Psychiatric symptoms were assessed by the 18-item version of Brief Psychiatric Rating Scale (BPRS) [18]. Items 1–10 are rated by the participant during an interview, while items 11–18 are rated by the researcher following observation of the participant. Adherence to medication over the past month was assessed with the following response options: (1) Nearly every day, (2) More than half the days, (3) About half the days, (4) Less than half the days, and (5) Not at all. [19] The economic burden of disease was self-assessed by items 1–6 of the Family Burden Scale of Disease (FBS) [20]. See Table S3 for the details of different scales.

### Covariates

Sociodemographic variables of patients including gender, age, duration of disease, years involved in the NMIPSMI program, location of residence, education level, marital status (married/single/divorced/widowed), household income, work status, whether living alone, and geographical accessibility of services were evaluated. See Table 1 for the details.

**Table 1:** Sociodemographic characteristics of the included patients (*n* = 972)

Variables	Total (*n* = 972)	Wuhan (195)	Changsha (*n* = 224)	Guangzhou (360)	Shenzhen (193)
**Gender**					
Male	446(45.9%)	94(48.2%)	104(46.4%)	160(44.4%)	88(45.5%)
Female	526(54.1%)	101(51.8%)	120(53.6%)	200(55.6%)	105(54.5%)
**Age** (year, Mean/ SD)	47.1 ± 13.3	43.5 ± 12.4	49.2 ± 13.2	49.8 ± 12.5	43.2 ± 14.2
**Duration of disease (year,** Mean/ SD**)**	19.1 ± 10.9	19.4 ± 10.2	20.3 ± 11.1	20.5 ± 11.0	14.8 ± 10.4
**Duration under management (year,** Mean/ SD**)**	7.1 ± 3.9	7.4 ± 4.2	7.6 ± 3.6	7.9 ± 3.7	4.7 ± 3.2
**Location of residence**					
Urban	806(82.9%)	123(63.1%)	130(58.0%)	360(100.0%)	193(100.0%)
Rural	166(17.1%)	72(36.9%)	94(42.0%)	0(0.0%)	0(0.0%)
**Education level**					
Primary	308(31.7%)	70(35.9%)	75(33.5%)	106(29.4%)	57(29.5%)
Middle or high	568(58.4%)	113(57.9%)	127(56.7%)	221(61.4%)	107(55.4%)
University or above	96(9.9%)	12(6.2%)	22(9.8%)	33(9.2%)	29(15.1%)
**Marital status**					
Single	365(37.6%)	78(40.0%)	71(31.7%)	139(38.6%)	77(39.9%)
Married / cohabited	491(50.5%)	91(46.7%)	127(56.7%)	180(50%)	93(48.2%)
Divorced	83(8.5%)	22(11.3%)	15(6.7%)	30(8.3%)	16(8.3%)
Widowed	33(3.4%)	4(2.0%)	11(4.9%)	11(3.1%)	7(3.6%)
**Family income below the poverty line**					
No	717(73.2%)	86(44.1%)	145(64.7%)	312(86.7%)	174(90.2%)
Yes	255(26.8%)	109(55.9%)	79(35.3%)	48(13.3%)	19(9.8%)
**Work status**					
Full-time	132(13.6%)	15(7.7%)	23(10.3%)	44(12.2%)	50(25.9%)
Part-time	31(3.2%)	3(1.5%)	4(1.8%)	9(2.5%)	15(7.8%)
Unemployed	706(72.6%)	170(87.2%)	181(80.8%)	246(68.3%)	109(56.5%)
Retired	103(10.6%)	7(3.6%)	16(7.1%)	61(17.0%)	19(9.8%)
**Living alone**					
No	868(89.3%)	176(90.3%)	202(90.2%)	316(87.8%)	174(90.2%)
Yes	104(10.7%)	19(9.7%)	22(9.8%)	44(12.2%)	19(9.8%)
**Geographical accessibility of services**					
< 15 min’ walk	344(35.4%)	94(48.2%)	72(32.1%)	82(22.8%)	96(49.7%)
16–30 min’ walk	125(12.9%)	29(14.9%)	37(16.5%)	28(7.8%)	31(16.1%)
< 15 min by car	214(22.0%)	24(12.3%)	44(19.6%)	102(28.3%)	44(22.8%)
16–30 min by car	241(24.8%)	47(24.1%)	58(25.9%)	115(31.9%)	21(10.9%)
> 30 min by car	48(4.9%)	1(0.5%)	13(5.9%)	33(9.2%)	1(0.5%)
**GAF (**Mean/ SD**)**	69.9 ± 18.3	65.6 ± 20.4	67.1 ± 18.1	68.9 ± 16.1	78.9 ± 16.9
**BPRS (**Mean/ SD**)**	22.6 ± 9.2	24.4 ± 13.3	22.8 ± 9.6	21.9 ± 6.8	21.7 ± 7.0
**WHO-DAS (**Mean/ SD**)**	24.8 ± 12.2	27.1 ± 13.4	26.2 ± 13.1	25.4 ± 11.3	19.6 ± 10.1
**Economic burden of disease (**Mean/ SD**)**	7.2 ± 4.2	7.5 ± 3.8	8.4 ± 3.9	7.5 ± 3.9	5.1 ± 4.4
**Quality of life (**Mean/ SD**)**	6.4 ± 1.3	6.4 ± 1.4	6.1 ± 1.2	6.5 ± 1.3	6.6 ± 1.5
**Medication adherence (**Mean/ SD**)**	4.5 ± 1.0	4.3 ± 1.2	4.4 ± 1.1	4.6 ± 0.8	4.6 ± 0.9

### Statistical analyses

The qualitative data of the included policies were grouped by DQ and YLL, where possible, into four analytical themes: (a) policies for basic public mental health services; (b) policies for social medical assistance; (c) policies for community-based rehabilitation; and (d) policies for risk management. Consensus on discrepancies in data extraction was reached through discussion.

SPSS 26.0 was used for the quantitative data analysis. All *p*-values refer to two-tailed tests. A *p*-value less than 0.05 was considered statistically significant. Continuous variables were described using mean and standard deviation, and categorical variables were described using frequency and percentage. Differences in the contact coverage of public mental health services between cities were examined by the single- factor chi-square test. Odds ratios and 95% confidence intervals were calculated to examine the associations between  the contact coverage of those public mental health services and different patient-related outcomes using multivariate linear regression analysis. The utilization rate of public mental health services was used as the dependent variable in different models, and some factors including gender, age, marital status, education level, family income, work status, live status, geographical accessibility of service, location of residence, duration of disease, years in the program were adjusted for in the models. See Table S4 for the details of the different models.

## Results

### Policies for public mental health services at the national level and in the four Southern cities

The national policies are mainly for basic public mental health services [10]. Health information technology (IT) systems for management of SMI have been publicized, and follow-up services, physical examinations, and violence risk assessments have been provided to patients. In addition, the government has mandated that 60% of outpatients should receive community-based rehabilitation services. For risk management, the government required that any patient responsible for an accident must receive compulsory medical treatment or hospitalization.

The four cities followed national guidelines in providing basic public mental health services [10]. Local health information technology (IT) systems and local quality control protocols were established for health information management in Guangzhou and Shenzhen. For social medical assistance services, they are mainly provided for local populations (people registered in the local household registration system) in the four cities. They can get free social medical insurance, allowances for outpatient service and inpatient services. For medication policies, there are some differences among the four cities. Patients who were Changsha residents can get essential medicines listed in the medical insurance for free, while local patients in Shenzhen or Wuhan can receive an allowance (¥150) for medication every month. For community-based rehabilitation services, the four cities require 50%-60% of outpatients to participate in these programs. For risk management, the policies for free emergency hospitalization were consistent – any patient responsible for an accident must receive compulsory medical treatment or hospitalization in the four cities. To promote community safety, the four cities have instituted local reward policies to encourage family care of individuals with SMI by providing financial incentives to the family. According to the documents, Guangzhou and Shenzhen offer higher bonuses than Wuhan. However, this policy is a non-public policy in Changsha, and the specific details are unknown. Additionally, Wuhan, Guangzhou, and Shenzhen have implemented local business insurance policies to reduce the risk of the patients’ family returning to poverty due to illness, and these cities have tried to purchase guardianship liability insurance for the guardians or accident insurance for patients with SMI. See Table 2 for the details.

**Table 2:** Policies for the public mental health services in China and the four cities

	**The central government**	**Wuhan**	**Changsha**	**Guangzhou**	**Shenzhen**
Basic public mental health services					
Health information management	National health IT systems are used for information management.	National health IT systems are used for information management.	National health IT systems are used for information management.	Local health IT systems and local quality control protocol were established.	Same as Guangzhou.
Follow-up services	Patients are followed-up every three months. The qualified management rate of registered patients with severe mental disorders should be over 80%.	Stable patients are followed up with every three months, while unstable patients are followed up every month.	Stable patients are followed up with every three months, while unstable patients are followed up every month.	Stable patients are followed up with every three months, while unstable patients are followed up every month.	Same as Guangzhou.
Physical examinations	1–2 physical examinations per year	1–2 physical examinations per year	1–2 physical examinations per year	1–2 physical examinations per year	Same as Guangzhou.
Violence Risk Assessments	Stable patients are followed-up every three months. Unstable patients are followed up every month.	Stable patients are followed up with every three months. Unstable patients are followed up every month.	Stable patients are followed up with every three months. Unstable patients are followed up every month.	Stable patients are followed up with every three months. Unstable patients are followed up every month.	Same as Guangzhou.
Social medical assistances					
Social medical insurance	NA	Purchase local social medical insurance for eligible patients.	Purchase local social medical insurance for eligible patients.	Purchase local social medical insurance for eligible patients.	Same as Guangzhou.
Medication	NA	Eligible underprivileged patients with a disability certificate released by the Wuhan government can receive an allowance (¥150 per month).	Patients who were Changsha residents can get essential medicines listed in the medical insurance for free.	NA	Eligible underprivileged patients with a disability certificate released by the Shenzhen government can get drug allowance (¥1,800 per year).
Outpatient service	NA	Local patients with a disability certificate (disability level greater than level 2) released by the Wuhan government. Underprivileged patients and eligible critically ill patients, can receive outpatient services with reimbursement up to ¥10,000 per year.	Local patients with social medical insurance, who are at risk of violence and have hospitalization records, can receive outpatient services with reimbursement up to ¥500 per month.	Local patients with a disability certificate (disability level greater than level 2) released by the Shenzhen government can receive outpatient services with reimbursement up to ¥1,000 per month.	Same as Guangzhou.
Inpatient service	NA	For out-of-pocket social medical expenses, reimbursement will be provided at a rate of 70%.	Local poor patients with social medical insurance and at risk of violence can receive inpatient services.	Local patients with a disability certificate (disability level greater than level 2) released by the Shenzhen government can receive inpatient services.	Same as Guangzhou.
Community-based Rehabilitation Services					
Policy	More than 60% of outpatients receive community rehabilitation services.	More than 60% of outpatients receive community rehabilitation services.	More than 60% of outpatients receive community rehabilitation services.	Establish at least one comprehensive community mental rehabilitation service center in each district.	More than 50% of outpatients receive community rehabilitation services.
Risk management					
Compulsory treatment	Any patient responsible for an accident must receive compulsory medical treatment or hospitalization.	Any patient responsible for an accident must receive compulsory medical treatment or hospitalization.	Any patient responsible for an accident must receive compulsory medical treatment or hospitalization.	Any patient responsible for an accident must receive compulsory medical treatment or hospitalization.	Same as Guangzhou.
Free Business insurance	NA	‘Guardianship Liability Insurance for Severe Mental Disorders’ are for guardians, and ‘patient accident insurance’ are available in two districts of Wuhan.	NA	The government has purchased a commercial insurance called ‘Guardianship Liability Insurance for Severe Mental Disorders’ for all guardians.	Same as Guangzhou.
Guardianship Grant	NA	Depending on the severity of the condition, guardians (residents) of people with severe mental disorders may receive guardianship benefits (the grants range from ¥2,400 to ¥4,200).	NA	Depending on the severity of the condition, guardians, and assistant guardians of people with severe mental disorders may receive guardianship benefits (the target coverage is 50%, the grants range from ¥1,500 to ¥6,000).	Same as Guangzhou.

### Resources of public mental health services among the included community health centers

The average number of psychiatrists per community health center was 0.2 in Wuhan, 0 in Changsha, 1.1in Guangzhou, and 0 in Shenzhen. The average number of psychologists per community health center was 0 in Wuhan, 0 in Changsha, 1.2 in Guangzhou, and 0.2 in Shenzhen. The average number of therapists per community health center was 0 in Wuhan, 0.8 in Changsha, 1.5 in Guangzhou, and 0.3 in Shenzhen, and the average number of nurses per community health center was 17.5 in Wuhan, 16.5 in Changsha, 22.8 in Guangzhou, and 13.8 in Shenzhen. The average number of beds per community health center was 24.1 in Wuhan, 12.1 in Changsha, 14.7 in Guangzhou, and 0 in Shenzhen. The average mental health budget per registered patient was ¥655.2 in Wuhan, ¥416.5 in Changsha, ¥1,288.4 in Guangzhou, and ¥2,704.3 in Shenzhen. Compared with other cities, the average registration rate per community health center was highest in Wuhan, while the average rate of high risk of violence (≥ level 3) among registered patients per community health center in Wuhan was lowest. The other indicators in the four cities were basically the same. See Table 3 for the details.

**Table 3:** Key indicators of public mental health services among the included community health centers

Key indicators in 2020	Wuhan (*n* = 9)	Changsha (*n* = 15)	Guangzhou (*n* = 15)	Shenzhen (*n* = 6)
Average number of psychiatrists per community health center	0.2	0	1.1	0.3
Average number of psychologists per community health center	0	0	1.2	0.2
Average number of nurses per community health center	17.5	16.5	22.8	13.8
Average number of beds per community health center	24.1	12.1	14.7	0
Average mental health budget per registered patient (¥)	655.2	416.5	1288.4	2704.3
**Basic public mental health services in 2020**				
Average registration rate per community health center	6.1‰	5.6‰	3.4‰	3.5‰
Average rate of being administered after registration per community health center	98.5%	98.4%	92.2%	94.0%
Average rate of standardized management per community health center	91.5%	98.4%	88.6%	94.2%
Average medication rate of registered patients per community health center	82.1%	91.5%	87.7%	90.4%
Average rate of high risk of violence (≥ level 3) among registered patients per community health center	0.9%	3.5%	2.3%	2.7%

### Sociodemographic characteristics of included patients

The mean age of the included patients was 47.1 years (SD: 13.3). The mean duration of mental disorders was 19.1 years (SD: 10.9). The mean duration under the management of community health centers was 7.1 years (SD: 3.9). 45.9% of the patients were male, and 82.9% lived in urban areas. Additionally, 31.7% of the patients had a primary education level, 13.6% were full-time workers, and 50.5% were married or cohabited. In addition, 26.8% of the patients reported that their family income was lower than the poverty line of their residence. Specific sociodemographic characteristics among patients in different cities were presented in Table 1.

### Contact coverage of public mental health services

Contact coverage of basic public mental health services in the four cities ranged from 56.7% to 93.3%, and the contact coverage of follow-up services in Changsha was significantly lower than in other cities (χ^2^** = **59.0 and 83.1; *p* < 0.05, respectively). No significant differences in the contact coverage of physical examination services were found (χ^2^** = **5.6; *p* > 0.05). The contact coverage of social medical assistance services in the four cities ranged from 10.9% to 97.8%. The contact coverage of free social medical insurance in Shenzhen was significantly lower than in other cities (χ^2^** = **37.3; *p* < 0.05). The contact coverage of medication assistance services in Changsha was significantly lower than in other cities (χ^2^** = **8.8; *p* < 0.05). Also, the contact coverage of outpatient services in Guangzhou was significantly higher than in other cities (χ^2^** = **408.8; *p* < 0.05). No significant differences in the contact coverage of outpatient services among the four cities were found (χ^2^** = **6.7; *p* > 0.05). In addition, the contact coverage of community-based rehabilitation services in the four cities ranged from 8.7% to 26.1%, and the contact coverage of community-based rehabilitation services in Guangzhou was significantly higher than in other cities (χ^2^** = **39.9; *p* < 0.05). See Table 4 for the details.

**Table 4:** Contact coverage of public mental health services from the perspective of registered patients in different cities

	**Coverage rate**				
	**Wuhan** **(*n* = 195)**	**Changsha (*n* = 224)**	**Guangzhou (*n* = 360)**	**Shenzhen** **(*n* = 193)**	**χ**^**2**^
**Basic public mental health services**					
Follow up service (≥ one time per year)	182(93.3%)	164(73.2%)	329(91.3%)	179(92.7%)	59.0*
Regular follow-up (≥ 4 times per year)	178(91.2%)	127(56.7%)	299(83.1%)	139(68.9%)	83.1*
Free physical examination (≥ one time per year)	153(78.5%)	193(86.1%)	301(83.6%)	166(86.0%)	5.6
**Social medical assistance services**					
Free social medical insurance	187(95.9%)	213(95.1%)	352(97.8%)	165(85.4%)	37.3*
Medication service	149(76.4%)	168(46.9%)	263(73.1%)	124(64.2%)	8.8*
Outpatient service	77(39.4%)	33(14.7%)	306(85.0%)	21(10.9%)	408.8*
Inpatient service	54(27.6%)	58(25.9%)	94(26.1%)	34(17.6%)	6.7
**Community-based rehabilitation**					
Community-based rehabilitation services	17(8.7%)	24(10.7%)	94(26.1%)	25(12.9%)	39.9*
**Risk management**					
Free emergency hospitalization	0(0.0%)	0(0.0%)	0(0.0%)	0(0.0%)	-
Free Business insurance^a^	NA	NA	360(100.0%)	193(100%)	-
Guardianship Grant for guardian ^b^	8/128(6.2%)	37/113(32.7%)	126/148(85.1%)	92/108(85.2%)	237.1*

^a^ the data were reported by the department responsible for this project

^b^ the data were reported by the patients’ guardian or family caregivers

^*^ *p* < 0.05

For risk management, none of the included patients were asked for emergency hospitalization over the past year, and the contact coverage of business insurance (guardianship liability insurance for guardians) in Guangzhou and Shenzhen was 100%. A total of 497 family members of the included patients were surveyed on the contact coverage of guardianship grants. The contact coverage of guardianship grants in Guangzhou and Shenzhen was significantly higher than that in Changsha and Wuhan (χ^2^** = **237.1; *p* < 0.05).

### Associations between contact coverage of public mental health services and outcomes of patients

The results showed that patients in Wuhan who received community-based rehabilitation services reported a lower level of disability (B**=**-6.5; 95%*CI:* -12.9 - -0.1). Patients in Changsha who received social medical assistance services reported a higher level of medication adherence (B**=**0.3; 95%*CI:* 0.1-0.5). Patients in Guangzhou who received community-based rehabilitation services reported better function (B**=**7.8; 95%*CI:* 4.1-11.6) and a lower level of disability (B**=**-2.8; 95%*CI:* -5.5 - -0.1), while those who received social medical assistance services reported a higher level of medication adherence (B**=**0.2; 95%*CI:* 0.1-0.3). In addition, patients in Shenzhen who received community-based rehabilitation services reported better function (B**=**6.3; 95%*CI:* 3.3-9.3), a lower level of disability (B**=**-2.6; 95%*CI:* -4.6 - -0.5), and a higher level of medication adherence (B**=**0.3; 95%*CI:* 0.1-0.4). Lastly, patients in Shenzhen who received social medical assistance services reported a higher level of medication adherence (B**=**0.2; 95%*CI:* 0.1-0.3). See Table 5 for the details.

**Table 5:** Associations between contact coverage of public mental health services and patients’ outcome

	**Wuhan** **(*n* = 195)**	**Changsha (*n* = 224)**	**Guangzhou (*n* = 360)**	**Shenzhen** **(*n* = 193)**
**GAF**				
Total number of contact coverages of basic public mental health services (0–2)	-2.9(-8.4–2.4)	2.5(-0.9–6.1)	-1.4(-4.2–1.3)	0.1(-1.6–1.9)
Total number of contact coverages of social medical assistance services (0–3)	-2.1(-5.8–1.4)	-2.2(-5.2–0.8)	1.4(-0.5–3.3)	-0.3(-1.5–0.9)
Community-based rehabilitation Services (No/Yes)	9.1(-0.8–19.4)	5.1(-2.4–12.7)	7.8(4.1–11.6) *	6.3(3.3–9.3) *
**BPRS**				
Total number of contact coverages of basic public mental health services (0–2)	0.5(-2.9–4.2)	-0.9(-2.9–1.0)	0.6(-0.6–1.0)	-0.1(-1.0–0.8)
Total number of contact coverages of social medical assistance services (0–3)	1.3(-3.3–3.8)	0.8(-0.9–2.4)	-0.8(-1.6–2.4)	-0.1(-0.8–0.5)
Community-based rehabilitation Services (No/Yes)	3.7(-1.1–10.5)	-0.4(-4.6–3.8)	-1.5(-3.2–3.8)	-1.0(-2.6–0.6)
**WHO-DAS**				
Total number of contact coverages of basic public mental health services (0–2)	3.2(-0.2–6.7)	1.1(-1.6–3.6)	2.58(1.6–4.5)	1.0(-0.2–2.2)
Total number of contact coverages of social medical assistance services (0–3)	2.6(-0.7–4.0)	0.5(-1.7–2.8)	-0.6(-1.9–0.8)	0.4(-0.5–1.2)
Community-based rehabilitation Services (No/Yes)	-6.5(-12.9–0.1) *	-4.2(-9.8–1.3)	-2.8(-5.5–0.1) *	-2.6(-4.6–0.5) *
**Economic burden of disease**				
Total number of contact coverages of basic public mental health services (0–2)	1.0(-0.1–1.9)	-0.1(-0.7–0.7)	0.1(-0.4–0.9)	0.1(-0.8–1.2)
Total number of contact coverages of social medical assistance services (0–3)	0.2(-0.8–0.5)	0.2(-0.3–0.9)	0.2(-0.3–0.6)	-0.4(-1.3–0.4)
Community-based rehabilitation services (No/Yes)	-2.3(-4.1—-0.5) *	-0.5(-2.1–1.1)	-0.7(-1.7–0.1)	1.2(-0.6–3.2)
**Quality of life**				
Total number of contact coverages of basic public mental health services (0–2)	-0.1(-0.2–0.5)	-0.1(-0.3–0.2)	-0.1(-0.3–0.1)	-0.1(-0.1–0.1)
Total number of contact coverages of social medical assistance services (0–3)	0.2(-0.3–0.2)	0.2(-0.1–0.4)	-0.1(-0.2–0.1)	0.1(-0.1–0.1)
Community-based rehabilitation Services (No/Yes)	-0.3(-0.5–0.9)	-0.3(-0.2–0.9)	0.3(-0.1–0.6)	0.2(-0.1–0.4)
**Medication adherence**				
Total number of contact coverages of basic public mental health services (0–2)	-0.2(-0.5–0.2)	0.1(-0.2–0.3)	-0.1(-0.1–0.1)	-0.1(-0.1–0.1)
Total number of contact coverages of social medical assistance services (0–3)	0.1(-0.1–0.3)	0.3(0.1–0.5) *	0.2(0.1–0.3) *	0.2(0.1–0.3) *
Community-based rehabilitation Services (No/Yes)	0.4(-0.2–0.9)	0.4(0.1–0.8)	0.2(-0.1–0.4)	0.3(0.1–0.4) *

^*^ *p* < 0.05

## Discussion

### Key findings

The results showed that the four cities in Southern China have made different efforts to develop public mental health services, primarily focusing on socioeconomically disadvantaged individuals. Some cities including Guangzhou and Shenzhen have released innovative risk management policies tailored to local needs. In addition, community health centers in Guangzhou and Shenzhen reported richer professional human resources on mental health and higher mental health budgets for the registered patients. The contact coverage rates of most public mental services were higher than 80%. Patients in Changsha, Guangzhou, and Shenzhen who received social medical assistance services reported higher levels of medication adherence. Meanwhile, patients in Wuhan, Guangzhou, and Shenzhen who received community-based rehabilitation services reported lower levels of disability.

### Comparisons with existing literature

In this study, community health centers in Guangzhou and Shenzhen (known as reform-driven coastal or eastern cities) reported professional human resources on mental health and higher mental health budgets for the registered patients than those in Wuhan or Changsha (relatively slow-developing central areas). In addition, relevant policies in Guangzhou and Shenzhen appear to be more comprehensive. For example, to improve the quality of health information management, they established local mental health IT systems and local quality control protocols. Furthermore, these two cities issued risk management policies tailored to local needs, several details of the policies are notable as very positive changes over recent years. In order to reduce the risk of patients’ families returning to poverty due to illness, the local governments and commercial insurance companies cooperated to develop exclusive commercial insurance for families with severe mental disorders—"Guardian Liability Compensation Insurance". Since 2017, the local governments in Guangzhou and Shenzhen have paid for guardianship liability compensation insurance for all legal guardians in their system. If a registered individual with severe mental illness causes or suffers an accident, their legal guardian can file a claim with the insurance company. It is said that the Chinese government is the only government in the world that has cooperated with commercial insurance companies to develop such kind of commercial insurance [21]. At present, more than 10 cities in China have formulated similar policies [21]. The NMIPSMI program has established an ongoing national database on persons with SMI, which has been helpful for commercial insurance companies working with the government to develop "Guardian Liability Compensation Insurance" [21]. For now, whether it is the government, commercial insurance companies, or families with severe mental disorders can be benefited from this policy, and it may be worthy of promotion across the developing countries.

To promote community safety, the four cities have instituted local reward policies to encourage family care of individuals with SMI by providing financial incentives to families. Despite concerns raised by some researchers a few years ago [22], these policy appear to be effective in the context of under-resourced mental health in primary health facilities [22]. Regardless of the eventual outcome, the lessons China has learned in closing the treatment gap will be useful to many other countries that are beginning to bridge the high burden of mental disorders. As China have tried many new ways to solve a common problem facing all countries, the world will also learn a lesson: finding the right balance between care and control [23]. In addition, we noted that most of the social medical assistance services were mainly provided to registered residents. However, some cities such as Shenzhen are immigrant cities, many patients were migrants [24]. Policy restrictions exclude most of these migrants from accessing services in the cities they live and work, even if they desperately need them [25]. To increase the coverage of public mental health services, we think governments in cities with large floating populations should change their policy provisions.

Previous studies reported that the lifetime prevalence of schizophrenia patients having contact with mental health-care providers or mental health services is 63.6% in China [26]. In this study, 98.1% (954/972) of patients reported that they have utilized at least one of the public mental health services over the past year (Table S5), which is higher than the data collected in 2001 (2.3%-10.0%) and 2015 (92.8%) [26]. In addition, the contact coverage of mental health services in this study was also higher than that in the Americas (13.9%-39.7%) [27]. Regardless of service quality, we believe that after more than 10 years of development, the NMIPSMI project has contributed to closing the treatment gap in China. Specifically, three of the services including follow-up services, physical examination services, and medication distribution services have a high utilization rate. While the other three services including inpatient services, outpatient services, and rehabilitation services have a lower utilization rate. One possible reason is that these three services with a lower utilization rate have participation restrictions. For example, in Changsha, if a patient chooses to participate in the medication services, he/she can no longer participate in the outpatient services. In addition, inpatient services are only available to those below the poverty line with violent behavior in those cities. We investigated the reasons why patients did not use these services (Table S5). Specifically, most of the patients (56.8%-92.2%) said they did not know the community health centers offered these services, and 0.5%-6.6% of patients said they knew these services were offered, but they were not eligible to participate. Only a few patients refused to participate in these services voluntarily. Therefore, it is evident to see that these services have problems in its implementation. Apart from restrictions in policies, these services may be poorly advertised by the providers. To improve the contact coverage of public mental health services among registered patients, future research is needed to further explore related barriers and propose solutions.

Patients in Wuhan, Guangzhou, and Shenzhen who received community-based rehabilitation services reported lower levels of disability. Patients in Changsha, Guangzhou, and Shenzhen who received social medical assistance services reported higher levels of medication adherence, which is consist with previous studies [19, 28]. Follow-up studies are needed to further explore the correlations.

### Limitations

Although this study found an association between service utilization and patient-related outcomes, causal inferences could not be made due to the cross-sectional design. For complex reasons, outcome evaluation was not incorporated into the initial design of these programs, making it methodologically challenging to conduct evaluation research [7]. Besides, researchers usually had no access to administrative data. To fully assess the outcomes of public mental health services, many hurdles need to be overcome in the future. To ensure the study was accessible to participants with a range of difficulties, travel expenses were covered (¥20). Efforts were made to include registered participants who were hospitalized. For individuals in the community, participants were personally invited through telephone calls. Despite these efforts, some patients refused to participate in the program, which may have affected the results. In addition, considering that the majority of registered patients (about 72–80%) have diagnosed schizophrenia [13, 29], and 18% have diagnosed mental retardation with psychotic symptoms due to epilepsy (who did not meet our inclusion criteria) [29], we only included patients with schizophrenia when collecting user-related data. Despite its low percentage (about 7%), registered patients with other three types of SMI (such as bipolar disorder) in the system might have different outcomes, which need further research. Also, considering that the patients included in this study were registered patients, it remains unclear whether unregistered patients would have different outcomes when using relevant services. Lastly, the research was conducted during the pandemic of COVID-19, which may have impact on the results.

### Future implications

Currently, less than 4% of the total health budget is spent on mental health care in China [5]. Most of the mental health expenditure still goes towards psychiatric hospitals, with community mental health care consistently underfunded [3]. In addition, previous research also indicated that a lot of treatment visits of people without need take place in (mostly reimbursed) formal healthcare settings [30]. Not only overtreatment but also suboptimal care may be regarded as wasting resources that prevent efficient mental healthcare [30]. Towards this end, budgets for public mental health care in China should be increased, and efforts are needed to ensure that the right treatment goes to the right people who need them. Besides, developing mental health services of good quality requires the use of evidence-based protocols [4]. Considering that the general medical professionals in primary care often lack training and resources for treating mental disorders [27], we are uncertain whether patients received services that adhere to evidence-based recommendations [14]. We believe the next step is to develop a comprehensive assessment framework to evaluate public mental health services in China. Another key to closing treatment gaps and improving patient outcomes in China is engaging service users, their family members, and supporters in the design, delivery, and evaluation of public mental health services [31]. Lastly, while the Chinese government has adopted audit-style performance accountability approaches in the health system, the possible adverse effects of performance targets on the development of public mental health services in low-resource primary health facilities should not be ignored [32]. Concerns on the quality of data which reported by service providers are needed to be clarified in the future.

## Conclusions

There have been advances in public mental health services in the four southern cities of China. The contact coverage rates of most public mental health services were higher than 80%. Patients’ utilization of public mental services was associated with better health outcomes. To improve the quality of public mental health services, the government should try to engage service users, their family members, and supporters in the design, delivery, operationalization, and evaluation of those public mental health services. Considering that no effective quality-control measures for public mental health services were implemented in most Chinese cities, and possible adverse effects of government performance targets, concerns on the quality of data which reported by service providers in these low-resource primary health facilities are needed to be clarified in the future.

### Supplementary Information

Supplementary Material 1.

## Data Availability

The datasets used and/or analysed during the current study available from the corresponding author on reasonable request.
