# Supplementary material for: Public mental health services in Southern China and related health outcomes among individuals living with severe mental illness
Source: Glob Health Res Policy. 2024 Aug 29;9:31. doi: 10.1186/s41256-024-00363-0 (PMC11363596; doi:10.1186/s41256-024-00363-0)
Supplement: Supplementary file 1 — Supplementary Material 1. [file 41256_2024_363_MOESM1_ESM.pdf]

Table S1 Sampling results for community health service centers

Administrative district	Numbers of community health service centers the district have	Numbers of community health service centers included
East Lake High-tech district, Wuhan	8	2
Xinzhou district, Wuhan	15	7
Furong district, Changsha	13	4
Changsha district, Changsha	23	11
Tianhe district, Guangzhou	26	8
Panyu district, Guangzhou	16	7
Luohu district, Shenzhen	10	3
Longgang district, Shenzhen	11	3

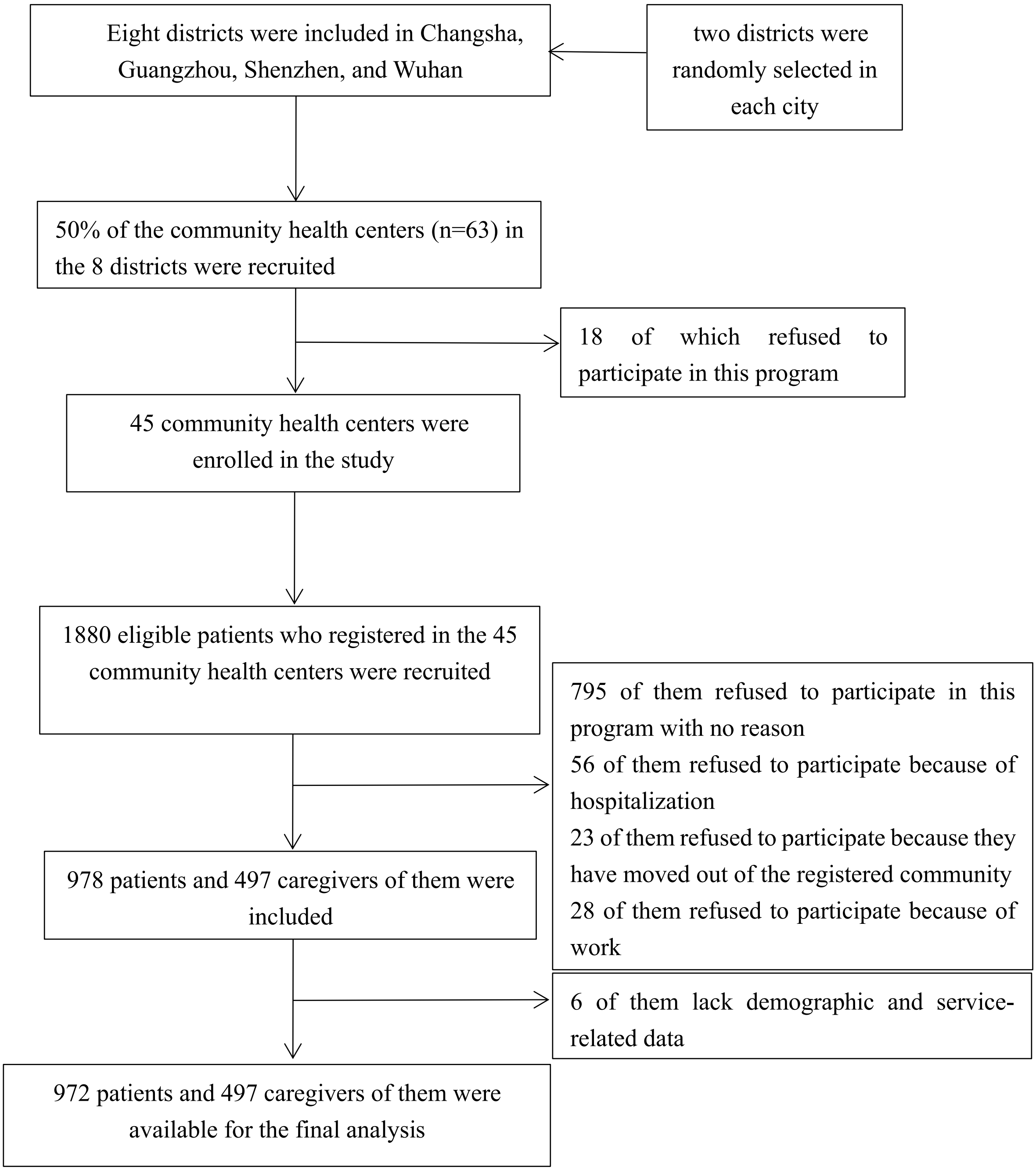

Fig. S1 Inclusion process for the participants

Table S2 inclusion criteria

**Policy/participants**	**Inclusion criteria / Exclusion criteria**
**Policies for public mental health services**	**Inclusion criteria:**<br>a) mental health policy released by the local government; b) other local policies included data for public mental health services.<br>**Exclusion criteria:** none
**Participants**	
Staff who reported policy-related data in each included city	**Inclusion criteria:**<br>a) work in the city mental health center; b) responsible for the work related to community mental health services<br>**Exclusion criteria:** none
Staff who reported policy-related data in each included district	**Inclusion criteria:**<br>a) work in the district mental health center; b) responsible for the work related to community mental health services
Staff who reported policy-related data in each included primary health centers	**Inclusion criteria:**<br>a) work in the community health center; b) responsible for the work related to mental health services<br>**Exclusion criteria:** none
Individuals with schizophrenia	**Inclusion criteria:**<br>a) adults over 18 years of age; b) with a diagnosis of schizophrenia by ICD-10; c) able to read and communicate<br>**Exclusion criteria:** a) Have epilepsy or severe head injury or serious head disease; b) have mental retardation
Family caregivers of the individuals with schizophrenia	**Inclusion criteria:**<br>a) adults over 18 years of age; b) a family member who is living with the patient and has taken the most responsibility of caring; c) able to read and communicate<br>**Exclusion criteria:** none

Table S3 The tools used for measurement of different variables

**Patient-related variables**	**Details of measurement**
Disability [1]	The WHODAS 2.0 as a general measure of functioning and disability in major life domains was used in this study. The WHODAS 2.0 was found to have high internal consistency (Cronbach's alpha, α: 0.86), a stable factor structure; high test-retest reliability (intraclass correlation coefficient:0.98). Items can be scored on a 5-point scale ranging from 1 = none

	to 5 = extreme/cannot do. The higher scores reflect greater disability.
Functioning [2, 3]	The Global Assessment of Functioning (GAF) was used to assess the patient's overall functioning and consists of one 100-point single item covering three major domains: social functioning, occupational functioning, and psychological functioning. The total score ranges from 1 to 100, with higher scores indicating higher overall functioning. Examples are given for each 10-level interval.
Quality of life [4]	Quality of life was measured using the first two general questions from the 14-item World Health Organization Quality of Life Brief Scale (WHOQOL-BREF), which is widely used across the world. The first item asks: "How do you evaluate your quality of life in the past two weeks?" on a 5-point scale from 1- "very bad" to 5- "very good". The second item asks: "Are you satisfied with your health status for the past two weeks?" on a 5-point scale from 1- "very unsatisfied" to 5- "very satisfied". Both items were self-rated by participants, with the total score ranging from 1 to 10 and a higher score indicating better quality of life.
Psychiatric symptoms [5, 6]	The Brief Psychiatric Rating Scale (BPRS) was used to assess the psychiatric symptomatology, including different symptoms, such as positive symptoms, negative symptoms, and affective symptoms, etc. The 18-item version of the scale was used in this study. Items 1–10 are rated by the participant during an interview, while items 11–18 are rated by the researcher following observation of the participant. Each item is rated on a 7-point scale anchored at 1=not present and 7=extremely severe.
Medication adherence [7]	Adherence to medication in the past months was assessed as follows: (1) Nearly every day. (2) More than half the days. (3) About half the days. (4) Less than half the days. (5) Not at all.
Economic burden of disease [8]	The family burden scale of disease (FBS) is a general questionnaire to measure the family burden of patients. There are 24 items in six dimensions in the FBS, which include family economic burden (six items), family daily activities (fix items), family entertainment activities (four items), family relationship (five items), physical health of family members (two items), and mental health of family members (two items). The first six items were used to economic burden of schizophrenia. The scale adopts three grades of 0–2, in which no burden has a rating of 0 and severe burden has a rating of 2.

Reference

1. Ustun, T.B., et al., *Developing the World Health Organization Disability Assessment Schedule 2.0.* Bull World Health Organ, 2010. **88**(11): p. 815-23.
2. Jones, S.H., et al., *A brief mental health outcome scale-reliability and validity of the Global Assessment of Functioning (GAF).* Br J Psychiatry, 1995. **166**(5): p. 654-9.
3. *Development of the World Health Organization WHOQOL-BREF quality of life assessment. The WHOQOL Group.* Psychol Med, 1998. **28**(3): p. 551-8.
4. Lodhi, F.S., et al., *Assessing the quality of life among Pakistani general population and their associated factors by using the World Health Organization's quality of life instrument (WHOQOL-BREF): a population based cross-sectional study.* Health and Quality of Life Outcomes, 2019. **17**.
5. Dazzi, F., A. Shafer, and M. Lauriola, *Meta-analysis of the Brief Psychiatric Rating Scale - Expanded (BPRS-E) structure and arguments for a new version.* J Psychiatr Res, 2016. **81**: p. 140-51.
6. Yu, Y., et al., *New Path to Recovery and Well-Being: Cross-Sectional Study on WeChat*

Use and Endorsement of WeChat-Based mHealth Among People Living With Schizophrenia in China. Journal of Medical Internet Research, 2020. **22**(9).
7. Gong, W.J., et al., *The association between a free medicine program and functioning in people with schizophrenia: a cross-sectional study in Liuyang, China.* Peerj, 2020. **8**.
8. Pai, S. and R.L. Kapur, *The burden on the family of a psychiatric patient: development of an interview schedule.* Br J Psychiatry, 1981. **138**: p. 332-5.

Table S4 Details of variables controlled by different linear regression models

Data Analysis	Model
**The associations between utilization rate of community-based public mental health services and patient-related outcomes (n=972)**<br>**Six different models were conducted for different outcomes**	**Outcome:** disability, functioning, psychiatric symptoms, quality of life, economic burden of disease, and medication adherence
	**Independent variable:** follow-up services<br>free physical examination service<br>rehabilitation Service<br>medicine distribution service<br>inpatient service<br>outpatient service<br>**Patient-related covariates:** gender, age, duration of schizophrenia, time under management, marital status, education level, family income, work status, living alone or not, location of residence, and geographical accessibility of service.

Table S5 Number of community-based mental health services received (n=972)

Number	Frequency	Percent (%)	Valid Percent (%)	Cumulative Percent (%)
0	18	1.79	1.79	1.9
1	58	6.0	6.0	7.8
2	210	21.6	21.6	29.4
3	281	28.9	28.9	58.3
4	272	28.0	28.0	86.3
5	110	11.3	11.3	97.6
6	23	2.4	2.4	100.0
**Total**	972	100.0	100.0	

Table S6 Reasons for not using the service

	N	I didn't know there was this service	I am not eligible to participate	I refuse to participate in this service	I don't need this service
**Medication service**	268	131(48.9%)	21(7.8%)	75(28.0%)	41(15.3%)
**Inpatient service**	732	675(92.2%)	28(3.8%)	7(1.0%)	22(3.0%)
**Outpatient service**	535	463(86.5%)	23(4.3%)	30(5.6%)	19(3.6%)
**Rehabilitation services**	811	619(76.3%)	4(0.5%)	170(21.0%)	18(2.2%)

Note: The program requires follow-up services and physical examination services for all patients under management, but the above four services were provided with

restrictions. Therefore, when designing the questionnaire, we only asked the reasons for not using the above four services.
